# Reprogramming the gastrointestinal microenvironment with intelligent biomaterials and digital medicine: a perspective on precision intervention for digestive diseases

**DOI:** 10.3389/fbioe.2026.1911713

**Published:** 2026-08-12

**Authors:** Linying Peng, Xueyan Jia, Huan Chen, Yanqiu Wang, Xiaoquan Du

**Affiliations:** 1 College of Traditional Chinese Medicine, Shaanxi University of Chinese Medicine, Xianyang, China; 2 Department of Gastroenterology, The Affiliated Hospital of Shaanxi University of Chinese Medicine, Xianyang, China

**Keywords:** artificial intelligence, digestive diseases, digital medicine, gastrointestinal microenvironment, inflammatory bowel disease, intelligent biomaterials, microbiome, mucosal barrier

## Abstract

Digestive diseases, including inflammatory bowel disease (IBD), gastrointestinal mucosal injury, colorectal cancer-associated inflammation, peptic ulcer disease, and postoperative gastrointestinal complications, are increasingly understood as disorders of a dynamic and spatially heterogeneous microenvironment rather than isolated abnormalities of a single tissue, cell type, or molecular pathway. The gastrointestinal microenvironment integrates epithelial barrier integrity, immune activation, microbiota composition, metabolic gradients, vascular remodeling, neural regulation, luminal pH, enzymatic activity, and mechanical stress. However, current clinical management remains largely organized around episodic diagnosis, systemic pharmacotherapy, endoscopic assessment, and reactive treatment escalation. This paradigm is insufficient for diseases characterized by relapsing inflammation, barrier disruption, microbiome instability, and patient-specific trajectories. Recent advances in intelligent biomaterials, artificial intelligence, digital health, and digital twin modeling create an opportunity to rethink gastrointestinal disease intervention as a process of microenvironmental reprogramming. In this Perspective, we propose that the next-generation of digestive disease care should move from disease suppression toward programmable regulation of the gastrointestinal microenvironment. Intelligent biomaterials are being investigated as locally acting therapeutic interfaces capable of responding to pH, enzymes, reactive oxygen species (ROS), inflammatory mediators, microbiota-derived metabolites, or tissue injury signals. Meanwhile, artificial intelligence (AI) and digital medicine are being developed to integrate endoscopic imaging, histology, multi-omics, symptoms, wearable data, microbiome profiles, and treatment history to support patient-specific decision-making. We argue that the key translational challenge is not simply to develop more advanced materials or more accurate algorithms in isolation, but to integrate digital interpretation with locally acting biomaterial interventions in clinically executable therapeutic systems. Using inflammatory bowel disease and related gastrointestinal disorders as representative contexts, this Perspective outlines current advances, conceptual gaps, and future directions for combining intelligent biomaterials and digital medicine to enable precision intervention in digestive diseases.

## 1 Introduction

Gastrointestinal diseases represent a major clinical and public health burden because they often combine chronic symptoms, recurrent tissue damage, systemic inflammation, impaired quality of life, and high long-term healthcare costs (Ehlers et al., 2020). Although conditions such as inflammatory bowel disease, colorectal cancer, gastrointestinal ulcers, radiation enteritis, postoperative anastomotic complications, intestinal fibrosis, and chemotherapy-induced mucositis are clinically diverse, many share a common pathological feature: disruption of the gastrointestinal microenvironment. This microenvironment is an active and spatially heterogeneous interface composed of the epithelial barrier, immune and stromal cells, endothelial cells, enteric neurons, microbiota, metabolites, mucus, extracellular matrix (ECM), digestive enzymes, and luminal physicochemical cues (Chang et al., 2022; Zhang et al., 2022).

Traditional treatment strategies have achieved important progress. Biologics, small-molecule immunomodulators, endoscopic surveillance, antibiotics, proton-pump inhibitors, surgical reconstruction, and nutritional interventions have improved outcomes for many patients (Kim et al., 2020; Njeim et al., 2025). Nevertheless, current clinical management remains limited by insufficient spatial control of localized mucosal lesions, reliance on episodic monitoring, heterogeneous therapeutic responses, and limited adaptation to dynamic changes in the gastrointestinal microenvironment. These limitations are particularly important in diseases characterized by relapsing inflammation, barrier disruption, microbiome instability, fibrosis, and patient-specific disease trajectories.

The rapid development of artificial intelligence, digital health, digital twin modeling, and advanced biomaterials provides a new opportunity to reframe digestive disease management. AI is already being explored in gastroenterology for endoscopic image interpretation, histological scoring, disease activity prediction, treatment response modeling, and clinical decision support (Lee and Kim, 2022). Recent reviews have highlighted the growing role of AI in inflammatory bowel disease diagnosis (Ren et al., 2025), classification, monitoring, and personalized care. At the same time, smart drug delivery systems and responsive biomaterials are being developed to release therapeutic agents in response to gastrointestinal pH, enzymes, inflammation-associated oxidative stress, microbiota-derived signals, or tissue injury. Together, these advances create the possibility of shifting digestive disease care from reactive disease suppression toward programmable regulation of local pathological microenvironments (Wang et al., 2025).

Despite these advances, the two fields often remain separated. Digital health research tends to focus on diagnosis, prediction, and monitoring (Weeda et al., 2022), whereas biomaterials research often focuses on material synthesis, drug loading, release behavior, and preclinical efficacy (Asa’ad et al., 2020). An integrated framework is therefore needed to connect digital interpretation with material-based therapeutic execution. In this framework, digital medicine may help identify clinically actionable gastrointestinal microenvironmental states, while intelligent biomaterials can provide local, responsive, and biologically instructive interventions. Innovation should not be defined only by better algorithms or better materials, but by the ability to translate patient-specific microenvironmental information into clinically executable local interventions.

This article proposes the concept of gastrointestinal microenvironment reprogramming as a translational framework. Here, gastrointestinal microenvironment reprogramming refers to a therapeutic strategy that actively reshapes disease-associated local conditions, including epithelial barrier disruption, immune activation, oxidative stress, microbiome imbalance, metabolic disturbance, fibrosis, and impaired tissue repair, through intelligent biomaterials guided by digital and patient-specific information. In this framework, digital medicine functions as the interpretive and adaptive layer. It integrates endoscopic imaging, histology, multi-omics, microbiome profiles, symptoms, wearable signals, and treatment history to define the patient-specific gastrointestinal microenvironmental state and guide therapeutic timing, location, and strategy. By contrast, intelligent biomaterials function as the local execution layer. They respond to disease-associated cues such as pH, enzymes, reactive oxygen species, inflammatory mediators, microbial metabolites, or tissue injury signals, and then deliver drugs, protective matrices, immunomodulatory cues, microbiome-regulating agents, or regenerative signals at diseased sites. Together, digital medicine and intelligent biomaterials provide a complementary strategy for data-guided, locally acting, and adaptive gastrointestinal intervention. This framework aligns closely with the mission of bioengineering and biotechnology because it emphasizes biomaterial design, tissue interaction, therapeutic function, preclinical validation, and clinical translation rather than purely computational prediction.

## 2 The gastrointestinal microenvironment as a programmable therapeutic target

The gastrointestinal tract is uniquely suited for microenvironment-oriented bioengineering because it contains multiple dynamic gradients and disease-associated signals (Carvalho et al., 2019). Unlike many solid organs, the gut is continuously exposed to luminal contents, microbial communities, digestive enzymes, bile acids, dietary components, mechanical peristalsis, mucus turnover, and epithelial renewal. In disease states, these features are altered in ways that may be exploited for targeted diagnosis and therapy.

### 2.1 Barrier disruption and epithelial repair

A healthy gastrointestinal barrier depends on intact epithelial tight junctions, mucus secretion, antimicrobial peptides, immune tolerance, and balanced microbiota-host interaction (Berkes et al., 2003). In inflammatory bowel disease and other inflammatory digestive conditions, barrier injury allows microbial products and luminal antigens to penetrate the mucosa, amplifying immune activation. In peptic ulcer disease, radiation injury, chemotherapy-induced mucositis, and postoperative intestinal injury, epithelial disruption also becomes a key driver of pain, bleeding, infection risk, and delayed healing.

From a biomaterials perspective, barrier dysfunction is not only a pathological feature but also a therapeutic opportunity. Adhesive hydrogels, mucoadhesive nanoparticles, injectable scaffolds, protective coatings, and epithelial-regenerative delivery systems may be designed to attach to injured mucosa, shield exposed tissue, release anti-inflammatory or pro-regenerative molecules, and support epithelial restitution. The goal is not simply to deliver drugs into the gastrointestinal tract, but to reconstruct a protective and instructive local niche (Zheng et al., 2025).

### 2.2 Immune imbalance and inflammatory amplification

Digestive diseases frequently involve dysregulated immune responses. In inflammatory bowel disease, inappropriate immune activation occurs in genetically susceptible individuals and is strongly influenced by microbial and environmental factors (de Lange et al., 2017). The gut microbiome is regarded as a major contributor to IBD pathogenesis, with dysbiosis often involving reduced bacterial diversity and changes in anti-inflammatory anaerobic bacteria (Chattopadhyay et al., 2022). In colorectal cancer, chronic inflammation can shape tumor immune microenvironments. In postoperative or ulcerative lesions, unresolved inflammation can delay tissue repair and increase complications.

Intelligent biomaterials may be engineered to modulate local immune states. For example, anti-inflammatory nanoparticles, ROS-scavenging polymers, macrophage-polarizing hydrogels, cytokine-sequestering materials, and microbiota-modulating delivery systems could reduce inflammatory amplification while preserving protective immunity (Bao et al., 2023). This is particularly important in the gut, where excessive immunosuppression may increase infection risk, whereas insufficient immune control may perpetuate tissue injury.

### 2.3 Microbiota, metabolites, and biochemical gradients

The microbiota is one of the defining features of the gastrointestinal microenvironment. It contributes to metabolism, immune education, epithelial function, and pathogen resistance. In disease, dysbiosis may alter short-chain fatty acid production, bile acid metabolism, mucin degradation, immune signaling, and epithelial energy balance (Lu et al., 2025). Therefore, digestive disease therapy should increasingly consider not only host cells but also microbial ecosystems.

This creates opportunities for biomaterials that deliver probiotics, postbiotics, antimicrobial peptides, bacteriophages, prebiotics, microbial metabolites, or microbiota-responsive drugs. Materials can be designed to respond to bacterial enzymes, inflammatory pH, oxidative stress, or colon-specific transit conditions. Such systems may allow more precise spatial and temporal regulation than conventional oral formulations.

### 2.4 Mechanical stress, fibrosis, and tissue remodeling

The gastrointestinal tract is mechanically active. Peristalsis, luminal pressure, ECM remodeling, and tissue stiffness influence disease progression and treatment response. In Crohn’s disease, for example, chronic transmural inflammation may lead to fibrosis and stricture formation (Small et al., 2013). In postoperative healing, mechanical stress at anastomotic sites can influence leakage risk and tissue repair (Sparreboom et al., 2016). In colorectal cancer, matrix stiffness and stromal remodeling affect invasion and drug penetration.

A microenvironment-reprogramming strategy should therefore include mechanical and structural dimensions. Bioadhesive materials, anti-fibrotic delivery systems, mechanically compliant hydrogels, and implantable or endoscopically delivered scaffolds may help regulate tissue repair, prevent pathological fibrosis, or reinforce vulnerable mucosal regions (Mostaghaci et al., 2017a). This is where gastrointestinal biomaterials can move beyond drug delivery toward tissue engineering and functional restoration.

## 3 Intelligent biomaterials as local executors of gastrointestinal microenvironment reprogramming

Intelligent biomaterials are materials designed to interact with biological systems in a responsive, instructive, or adaptive manner. For gastrointestinal diseases, their value lies in their ability to overcome several limitations of systemic therapy: poor local concentration, off-target toxicity, degradation in the digestive tract, variable absorption, and limited responsiveness to disease activity (Gutiérrez and Domingo-Calap, 2020).

### 3.1 pH-responsive and enzyme-responsive delivery systems

The gastrointestinal tract contains strong pH gradients, from acidic gastric conditions to near-neutral or slightly alkaline intestinal environments. Diseased tissues may also display altered pH due to inflammation, ischemia, infection, or tumor metabolism. These gradients can be used to design site-specific delivery systems. Enteric coatings, pH-sensitive polymers, acid-degradable nanoparticles, colon-targeted capsules, and hydrogel carriers have all been explored to protect therapeutic agents during transit and release them at desired locations (Gvozdeva and Staynova, 2025; Zhang et al., 2025).

Enzyme-responsive systems are particularly attractive because intestinal inflammation and microbial dysbiosis can alter local enzyme activity. Recent reviews emphasize that enzyme-responsive polymeric drug delivery platforms may enable site-specific and controlled release in the pathological microenvironment of inflammatory bowel disease (Rabiee et al., 2019). Compared with simple pH-triggered release, enzyme-responsive systems may better reflect disease-associated biological activity.

However, there is a key translational challenge. Most preclinical studies evaluate release behavior under simplified *in vitro* conditions, but the human gastrointestinal environment varies widely among patients, disease stages, diets, microbiota compositions, medications, and transit times. Therefore, future material design should move from generic responsiveness toward patient-stratified responsiveness.

### 3.2 ROS-responsive and inflammation-responsive materials

ROS are closely linked to mucosal inflammation, epithelial injury, and immune activation (Mittal et al., 2014). In inflammatory digestive diseases, excessive ROS can damage epithelial cells, proteins, lipids, DNA, and extracellular matrix (Chanin et al., 2020). ROS-responsive materials can be designed either to release anti-inflammatory drugs in oxidative environments or to directly scavenge ROS. Such materials may help shift the local microenvironment from destructive inflammation toward repair (Qiao et al., 2023).

A key perspective is that ROS should not be treated only as a trigger for drug release. It is also a pathological mediator and a measurable disease signal. Therefore, ROS-responsive materials may serve dual functions: sensing disease activity and executing local therapy (Xin et al., 2025). In future systems, ROS-sensitive nanoparticles or hydrogels could potentially be paired with imaging, biosensing, or digital monitoring platforms to provide information about inflammatory burden and therapeutic response.

### 3.3 Mucoadhesive and lesion-targeting biomaterials

One of the major obstacles in gastrointestinal therapy is retention. Oral drugs and nanoparticles must survive gastric acid, enzymatic degradation, mucus turnover, peristalsis, and fecal clearance. Mucoadhesive materials can prolong residence time by interacting with mucins or exposed epithelial surfaces (Das et al., 2025). Lesion-targeting materials may exploit charge interactions, inflammation-associated proteins, ulcerated mucosa, immune cell recruitment, or microbial biofilms.

For ulcerative lesions, hydrogels and nanoparticles that adhere preferentially to damaged mucosa may provide a local therapeutic depot (Dobre et al., 2018). For colorectal lesions, materials that accumulate in inflamed or tumor-associated regions may improve drug concentration and reduce systemic exposure. For postoperative intestinal injury, sealant-like biomaterials may support tissue closure while delivering anti-inflammatory, antimicrobial, or pro-angiogenic factors (Avalos-González et al., 2010).

### 3.4 Biomaterials for microbiome modulation

The microbiome is both a therapeutic target and a therapeutic partner. Conventional antibiotics can disrupt microbial ecosystems, whereas probiotics and fecal microbiota transplantation remain difficult to standardize. Biomaterials may offer more controllable strategies, including encapsulation of live bacteria, protection of probiotics from gastric acid, localized release of prebiotics, delivery of microbial metabolites, or selective modulation of pathogenic bacteria (Novak et al., 2009).

In this context, the goal is not simply to “restore the microbiome” in a general sense. A more precise goal is to reshape microbial functions relevant to barrier repair, inflammation resolution, metabolic balance, and pathogen resistance. AI-guided microbiome analysis and multi-omic modeling may help identify which microbial functions should be targeted in specific patient subgroups (Kadan et al., 2025). A recent real-world study has explored AI-guided multi-omic microbiome modulation in IBD, reflecting the broader trend toward data-informed microbiome intervention.

### 3.5 Tissue-regenerative and anti-fibrotic biomaterials

For digestive diseases, tissue repair is as important as inflammation control. Chronic mucosal injury can lead to ulceration, bleeding, strictures, fistulas, and impaired absorptive function. Regenerative biomaterials can deliver growth factors, extracellular vesicles, nucleic acids, peptides, stem-cell-derived products, or matrix-mimetic cues to promote epithelial repair and stromal remodeling (Pham et al., 2023).

However, regeneration in the gut must be carefully controlled. Excessive fibrosis, abnormal angiogenesis, or dysregulated epithelial proliferation could worsen disease or increase neoplastic risk (Buret, 2007). Therefore, gastrointestinal regenerative biomaterials should be designed with disease-specific biological endpoints: barrier restoration, inflammation resolution, controlled matrix remodeling, vascular normalization, and prevention of pathological fibrosis. These examples indicate that intelligent biomaterials can execute local microenvironmental regulation, but material selection alone is insufficient without a reliable method for identifying which pathological state predominates in a given patient. This need creates the rationale for digital medicine as the interpretive layer that links patient-specific data to biomaterial-based intervention.

## 4 Digital medicine as the interpretive layer for precision gastrointestinal intervention

Building on the local therapeutic functions of intelligent biomaterials, digital medicine provides the interpretive layer needed to determine when, where, and for whom these material-based interventions should be applied. If intelligent biomaterials are the local executors of microenvironmental therapy, digital medicine can be viewed as the interpretive layer that determines patient state, therapeutic timing, risk trajectory, and response evaluation. This relationship shifts the discussion from isolated AI-based diagnosis toward data-guided therapeutic decision-making. The central issue is therefore not simply whether digital tools can predict disease activity, but how they can guide material-based interventions toward the most actionable gastrointestinal microenvironmental features.

### 4.1 AI-assisted endoscopy and histology

Endoscopy is central to digestive disease diagnosis and monitoring. AI systems have been widely explored for lesion detection, polyp recognition, inflammatory activity scoring, dysplasia detection, and quality control. In inflammatory bowel disease, AI applications are being investigated in endoscopy, histology, diagnosis, monitoring, treatment response prediction, and personalized care.

For microenvironment reprogramming, AI-assisted endoscopy has a specific value: it can help localize disease regions that may benefit from topical, endoscopic, or region-specific biomaterial therapy. For example, AI-based segmentation of ulcerated mucosa could guide where a hydrogel should be sprayed, where a drug-eluting patch should be placed, or where biopsy and microbiome sampling should be performed (Athab et al., 2025). In the future, endoscopic imaging may not only diagnose lesions but also guide local bioengineering interventions (Wu et al., 2025).

### 4.2 Digital biomarkers and longitudinal disease monitoring

Digestive disease activity is often dynamic. Symptoms may not correlate perfectly with mucosal inflammation. Laboratory markers may lack spatial resolution. Endoscopy is informative but invasive and episodic. Digital biomarkers may help fill this gap by integrating patient-reported outcomes, wearable data, stool characteristics, diet records, medication adherence, sleep, physical activity, inflammatory markers, microbiome profiles, and imaging history (Bonham, 2018). Recent advancements in Internet of Things (IoT)-based frameworks combined with attention-based deep learning further enable continuous, multi-disease monitoring systems that could be adapted for dynamic gastrointestinal tracking (Nainwal et al., 2025).

For IBD, recent work has emphasized the potential of digital biomarkers and AI to support diagnosis, monitoring, and treatment (Zhang et al., 2019). For biomaterial-guided therapy, such biomarkers could help determine whether a patient needs anti-inflammatory release, barrier-protective intervention, microbiome modulation, or fibrotic remodeling therapy. In this sense, digital biomarkers could become the “control signals” for future adaptive gastrointestinal interventions.

### 4.3 Digital twins for digestive disease trajectories

Digital twin models aim to create patient-specific computational representations that can simulate disease evolution and therapeutic response. In precision medicine, digital twins are increasingly discussed as tools enabled by data collection, AI, and mechanistic modeling (Asciak et al., 2025). However, experts have argued that hypothesis-driven, multiscale mechanistic models are important to avoid overreliance on black-box prediction.

For gastrointestinal diseases, digital twin thinking is especially relevant because disease behavior depends on interacting biological, microbial, mechanical, immune, dietary, and treatment-related factors. A colorectal digital twin concept has recently been proposed for proactive and personalized management of colorectal cancer, inflammatory bowel disease, and diverticular disease, integrating multimodal physiological and behavioral data with hybrid mechanistic-machine learning modeling (Lin et al., 2023).

From the perspective of intelligent biomaterials, digital twins could help answer practical translational questions. Which patients are likely to benefit from local anti-inflammatory therapy? Which lesions require mucosal protection rather than systemic escalation? Which microbiome features suggest a need for targeted microbial modulation? Which postoperative patients are at high risk of anastomotic complications and may benefit from local protective materials? These questions are more clinically actionable than simply predicting disease activity.

### 4.4 Data-guided patient stratification for biomaterial therapy

A major weakness in current biomaterial studies is that they often assume one material strategy will apply broadly to an entire disease category. However, inflammatory bowel disease, colorectal cancer, gastrointestinal ulcers, and postoperative complications are highly heterogeneous. Patients differ in lesion location, immune phenotype, microbiome state, barrier function, drug exposure history, fibrosis risk, nutritional status, and comorbidities. AI may help stratify patients into clinically meaningful microenvironmental phenotypes. These phenotypes are intended to describe dominant and actionable pathological states rather than fixed disease labels. These phenotypes may include inflammation-dominant, barrier-disruption-dominant, dysbiosis-dominant, fibrosis-prone, oxidative-stress-dominant, poor-healing postoperative, or tumor-associated inflammatory states. Briefly, an inflammation-dominant phenotype refers to persistent immune activation, inflammatory cytokine elevation, and mucosal inflammatory injury. A barrier-disruption-dominant phenotype emphasizes epithelial damage, impaired tight junction integrity, mucus layer disruption, and increased mucosal permeability. A dysbiosis-dominant phenotype reflects microbial imbalance, altered microbial metabolites, and disturbed host–microbiome interactions. A fibrosis-prone phenotype indicates stromal activation, extracellular matrix remodeling, tissue stiffening, and potential stricture formation. An oxidative-stress-dominant phenotype refers to excessive reactive oxygen species production and redox imbalance. A poor-healing postoperative phenotype may involve ischemia, bacterial contamination, mechanical tension, and delayed tissue repair. A tumor-associated inflammatory phenotype is characterized by interactions among tumor cells, stromal remodeling, immune suppression, microbiota, and inflammatory signaling. Because these phenotypes represent different dominant pathological processes, each state may require a distinct material design and therapeutic function (Table 1). For example, an inflammation-dominant phenotype may benefit from locally retained anti-inflammatory or cytokine-modulating biomaterials (Helmecke et al., 2022). By contrast, a barrier-disruption-dominant phenotype may require mucoadhesive protective hydrogels, epithelial-regenerative carriers, or barrier-restoring coatings. A dysbiosis-dominant phenotype may be more suitable for probiotic encapsulation, prebiotic delivery, postbiotic release, or microbiome-responsive systems. A fibrosis-prone phenotype may require materials capable of regulating ECM deposition, stromal activation, and tissue remodeling. In this sense, digital medicine and biomaterials become mutually dependent: AI identifies the therapeutic context, while biomaterials execute the local intervention. The next step is to organize this relationship into a clinically executable sequence that begins with microenvironmental sensing, proceeds through computational interpretation and biomaterial selection, and ends with feedback-based adjustment after treatment.

**TABLE 1:** Microenvironmental features, digital readouts, and biomaterial strategies in digestive diseases.

Microenvironmental feature	Representative disease context	Digital/clinical readouts	Biomaterial strategy	Therapeutic goal
Barrier disruption	IBD, ulcers, mucositis	Endoscopy, histology, fecal biomarkers	Mucoadhesive hydrogels, epithelial repair carriers	Restore mucosal barrier
Excessive ROS	IBD, radiation enteritis	Inflammatory markers, imaging, tissue assays	ROS-responsive/scavenging materials	Reduce oxidative injury
Dysbiosis	IBD, postoperative infection	Microbiome sequencing, metabolomics	Probiotic encapsulation, prebiotic delivery	Rebalance microbiota
Enzyme-rich inflammation	Colitis	Fecal enzymes, tissue biomarkers	Enzyme-responsive polymers	Site-specific drug release
Fibrosis/remodeling	Crohn’s disease, strictures	Imaging, endoscopy, stiffness indicators	Anti-fibrotic delivery systems	Prevent pathological remodeling
Poor surgical healing	Anastomosis, fistula	Risk models, imaging, clinical monitoring	Sealants, antimicrobial hydrogels	Improve local healing

## 5 A proposed framework: from microenvironment sensing to adaptive intervention

**FIGURE 1:**
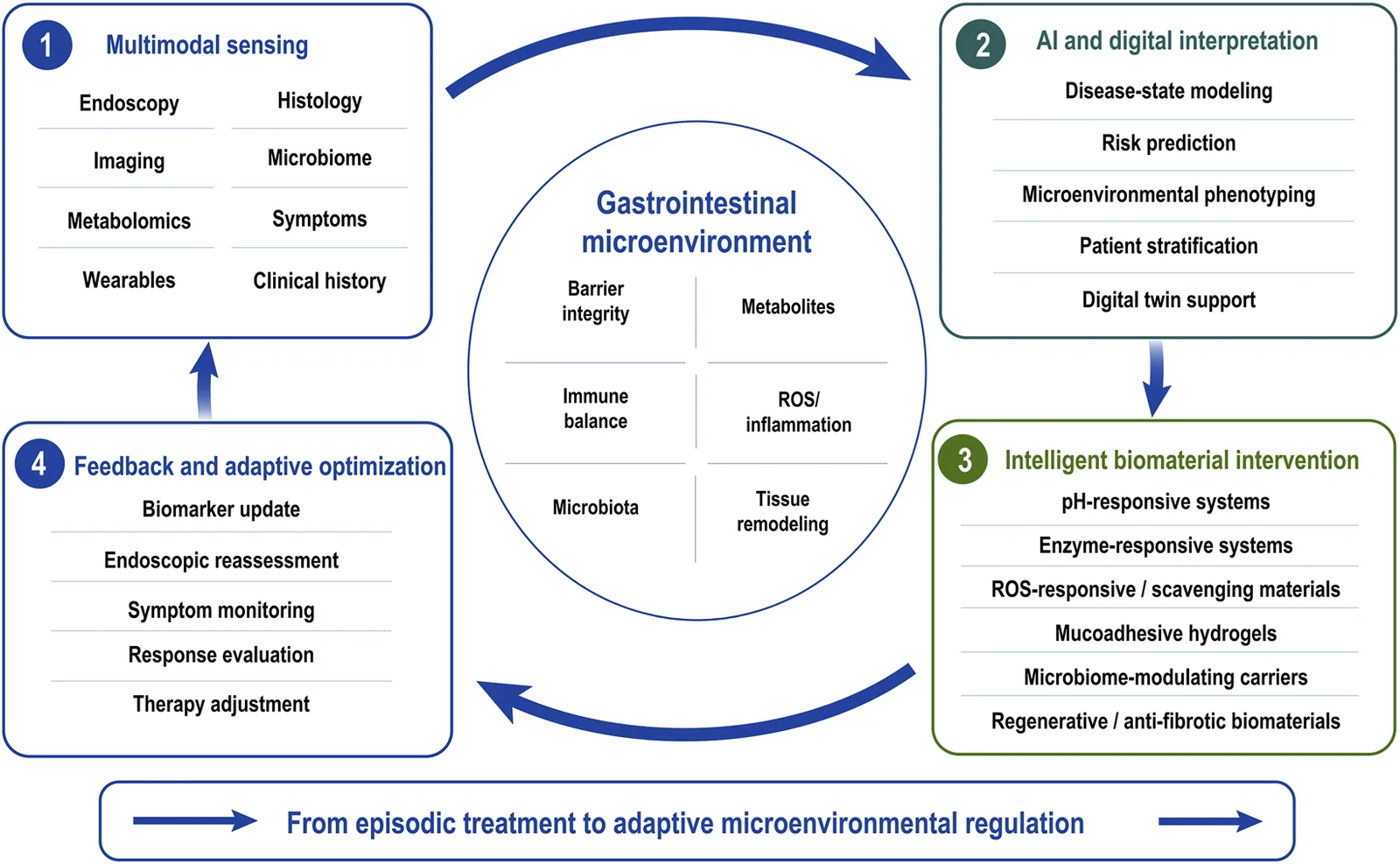
Conceptual framework of gastrointestinal microenvironment reprogramming with intelligent biomaterials and digital medicine.

Based on the complementary relationship between digital interpretation and biomaterial execution, we propose a four-layer framework for precision intervention in digestive diseases (Figure 1). This framework is designed to translate microenvironmental information into adaptive treatment decisions by connecting disease-state acquisition, computational interpretation, intelligent biomaterial-based intervention, and post-treatment feedback.

### 5.1 Layer 1: multimodal disease-state acquisition

The first layer is data acquisition. It includes endoscopy, histology, radiology, blood tests, fecal biomarkers, microbiome sequencing, metabolomics, patient-reported symptoms, wearable data, diet records, and medication history. The purpose is not to accumulate data for its own sake, but to define the patient’s gastrointestinal microenvironmental state.

For example, a patient with Crohn’s disease may have moderate symptoms but severe ileal ulceration, high fecal calprotectin, dysbiosis, and early fibrotic narrowing. Another patient may have frequent symptoms but minimal inflammation and a microbiome-metabolic disturbance. These patients should not receive identical material-based interventions.

### 5.2 Layer 2: computational interpretation and patient stratification

The second layer uses AI and mechanistic modeling to convert multimodal data into interpretable disease states. Importantly, the output should not only be a diagnostic label. It should identify actionable microenvironmental features: inflammation intensity, barrier damage, microbial imbalance, oxidative stress, fibrosis risk, tumor-associated immune state, or postoperative healing risk.

This layer should also include uncertainty estimation. In clinical translation, an AI model that predicts disease severity without communicating uncertainty may be difficult to trust. For biomaterial therapy, uncertainty is especially important because local delivery systems may involve invasive or semi-invasive administration.

### 5.3 Layer 3: intelligent biomaterial-based intervention

The third layer is the therapeutic execution layer. Depending on the disease state, intervention may involve pH-responsive oral nanoparticles for colonic drug release, enzyme-responsive systems for inflamed intestinal tissue, ROS-scavenging hydrogels for ulcerated mucosa. Other strategies may include mucoadhesive coatings for barrier protection, probiotic-encapsulating biomaterials for microbiome modulation, anti-fibrotic delivery systems for stricture-prone disease, and endoscopically sprayed hydrogels for mucosal repair. Local immunomodulatory depots for postoperative healing, or tumor microenvironment-responsive carriers for colorectal cancer therapy. The key requirement is that material selection should be justified by microenvironmental phenotype rather than by disease name alone. For instance, two patients diagnosed with inflammatory bowel disease may require completely different material strategies. One patient may be characterized mainly by superficial mucosal ulceration and oxidative stress. Another may be dominated by transmural fibrosis and microbiome instability. Therefore, the future of gastrointestinal biomaterials should move from disease-category-based design toward microenvironment-state-based design.

### 5.4 Layer 4: feedback, adaptation, and outcome learning

The fourth layer is feedback. After intervention, digital biomarkers, symptoms, laboratory markers, imaging, endoscopy, and microbiome data can be used to evaluate whether the microenvironment has shifted toward resolution. This creates an adaptive loop. If inflammation decreases but barrier repair remains incomplete, the next intervention may focus on epithelial regeneration. If inflammation improves but fibrosis progresses, anti-fibrotic strategies may be needed. If microbiome instability persists, microbial modulation may be added.

This framework does not require immediate deployment of a fully autonomous closed-loop system. Instead, it proposes a practical translational direction: digestive disease treatment should gradually become more data-guided, local, adaptive, and microenvironment-oriented.

## 6 Representative disease contexts

**FIGURE 2:**
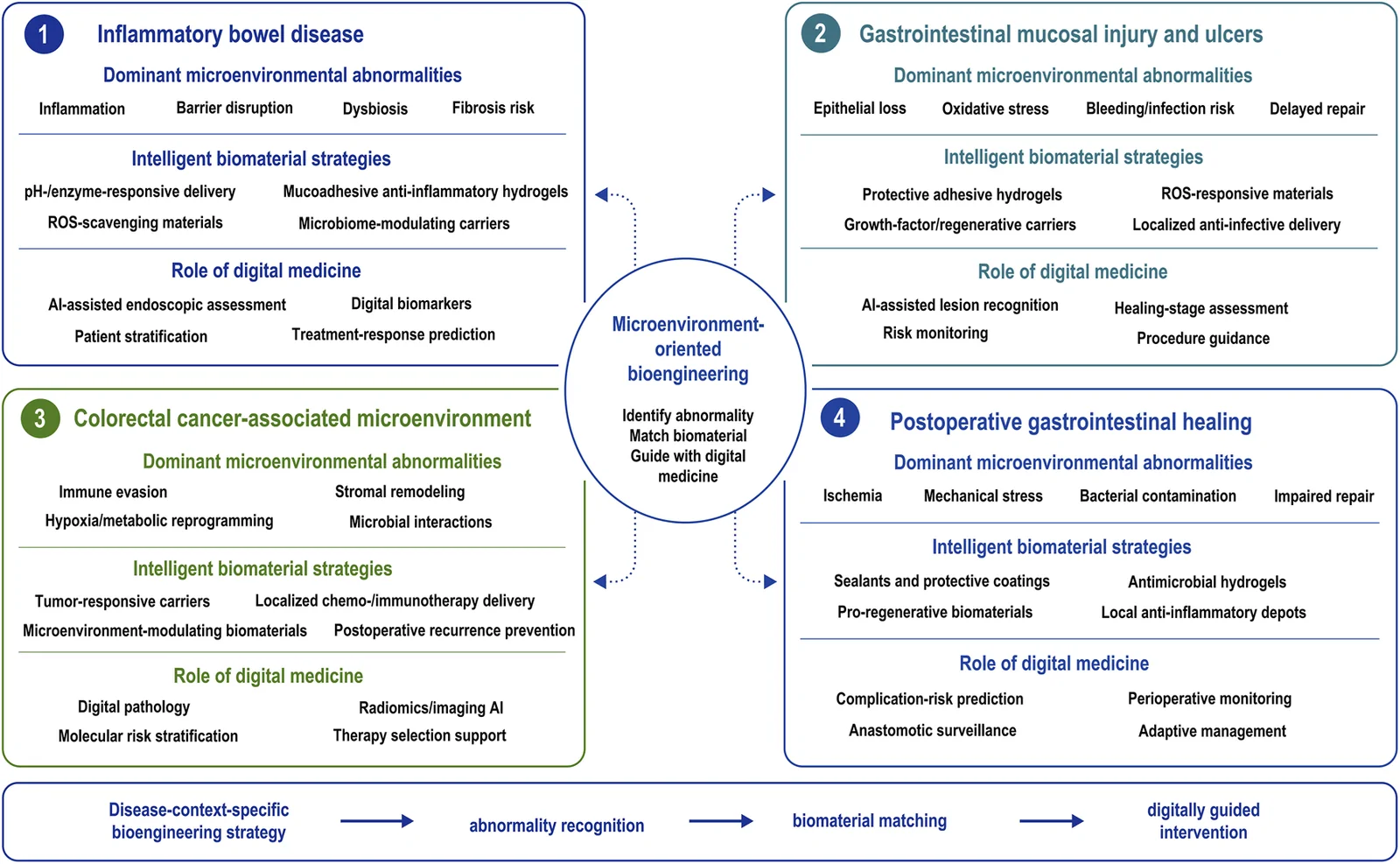
Representative gastrointestinal disease contexts for microenvironment-oriented bioengineering.

To illustrate the clinical translation of this framework, we highlight several representative gastrointestinal diseases contexts using a consistent structure: dominant microenvironmental features, digital interpretation strategies, biomaterial-based interventions, and translational implications (Figure 2).

### 6.1 Inflammatory bowel disease

Inflammatory bowel disease is an ideal representative context because its microenvironment is characterized by recurrent mucosal inflammation, epithelial barrier dysfunction, microbiome dysbiosis, immune imbalance, oxidative stress, and heterogeneous treatment response. From the digital medicine perspective, AI has been increasingly investigated for endoscopic assessment, histological interpretation, activity prediction, and treatment selection (Iacucci et al., 2025). Smart biomaterials are being investigated to support local anti-inflammatory delivery, barrier repair, microbiome modulation, and fibrosis prevention.

The current challenge is that these advances are often evaluated separately. Future IBD innovation should connect disease phenotyping with material selection. For example, a patient with distal ulcerative colitis and superficial mucosal ulceration may benefit from mucoadhesive anti-inflammatory hydrogels or rectal delivery systems (Wu et al., 2025). A patient with ileal Crohn’s disease and fibrotic progression may require anti-fibrotic and tissue-remodeling strategies. A patient with dysbiosis-dominant relapse may require microbiome-targeted biomaterials. Thus, IBD illustrates how the same diagnostic category may require different biomaterial strategies when the dominant microenvironmental state differs.

### 6.2 Gastrointestinal mucosal injury and ulcer disease

Gastrointestinal mucosal injury and ulcer disease include peptic ulcer disease, drug-induced injury, radiation enteritis, chemotherapy-induced mucositis, and ischemic intestinal damage. Their dominant microenvironmental features include epithelial loss, exposed extracellular matrix, oxidative stress, inflammatory amplification, microbial invasion, bleeding risk, and impaired mucosal repair. From the digital medicine perspective, endoscopic imaging, histology, clinical symptoms, laboratory markers, and recurrence history may help evaluate ulcer depth, inflammatory burden, bleeding risk, and healing status. From the biomaterial perspective, intelligent biomaterials may protect exposed mucosa, neutralize harmful microenvironmental signals, deliver growth-promoting factors, reduce infection risk, and support epithelial restitution.

The translational implication is that mucosal injury provides a clinically accessible setting for image-guided local therapy. For example, AI-assisted endoscopy could identify ulcer depth, bleeding risk, or healing status, while local biomaterial therapy could be applied during the same procedure (Bossuyt et al., 2020). This disease context highlights the value of integrating lesion localization with topical, adhesive, or regenerative biomaterial strategies.

### 6.3 Colorectal cancer-associated microenvironment

Colorectal cancer is not only a genetic disease but also a microenvironmental disease. Its dominant microenvironmental features include immune evasion, stromal remodeling, microbial interactions, hypoxia, metabolic reprogramming, angiogenesis, and barrier dysfunction. From the digital medicine perspective, endoscopic AI, digital pathology, radiomics, molecular profiling, and microbiome analysis may help characterize tumor location, invasion pattern, immune contexture, stromal status, and patient-specific recurrence risk. From the biomaterial perspective, biomaterials have been investigated for local chemotherapy, immunotherapy delivery, photothermal or photodynamic strategies, postoperative recurrence prevention, and tumor microenvironment modulation (Kang et al., 2025).

The translational implication is that colorectal cancer-associated biomaterial therapy should remain grounded in the gastrointestinal-specific tumor niche. Unlike many solid tumors, colorectal cancer develops within a microbiota-rich, mucus-covered, mechanically active, and immune-regulated intestinal niche. Therefore, digital pathology, endoscopic AI, radiomics, molecular profiling, and microbiome analysis may help identify which patients are most suitable for local biomaterial-assisted therapy (Reddy and Nelakuditi, 2025).

### 6.4 Postoperative gastrointestinal healing

Postoperative gastrointestinal healing is a representative context in which local microenvironmental failure can directly lead to clinical complications. After gastrointestinal surgery, complications such as anastomotic leakage, infection, delayed healing, fibrosis, and stricture formation remain clinically important. The dominant microenvironmental features at the surgical site include ischemia, inflammatory activation, bacterial contamination, mechanical tension, matrix remodeling, impaired vascularization, and delayed epithelial repair. From the digital medicine perspective, perioperative risk models, imaging, laboratory markers, surgical records, and postoperative monitoring may help identify patients at high risk of leakage, infection, or poor healing. From the biomaterial perspective, biomaterials have been explored as sealants, antimicrobial coatings, growth factor carriers, anti-inflammatory depots, or mechanically supportive interfaces (Byrne et al., 2021).

The translational implication is that postoperative gastrointestinal healing may benefit from risk-adapted local reinforcement. A future direction is to use patient-specific risk models to determine when local biomaterial reinforcement is needed and which biological function the material should provide, such as sealing, antimicrobial protection, vascular support, inflammation control, or anti-fibrotic remodeling.

## 7 Translational barriers and design principles

### 7.1 Moving beyond material performance in simplified models

Many gastrointestinal biomaterial studies demonstrate promising drug release, biocompatibility, anti-inflammatory effects, or tissue repair *in vitro* or in small animal models (Mostaghaci et al., 2017b). However, translation requires more than material performance. The material must function under complex gastrointestinal conditions: mucus turnover, enzyme degradation, microbial metabolism, variable pH, peristalsis, food interaction, and disease heterogeneity (Sohal et al., 2018).

Therefore, future studies should include more realistic models, such as organoids, gut-on-a-chip systems, *ex vivo* mucosal tissues, microbiota-containing models, and disease-relevant animal models. Evaluation should include retention, degradation products, immune response, microbiome effects, barrier function, fibrosis, toxicity, and long-term safety.

### 7.2 Designing materials around clinical workflows

For clinical translation, the route of administration is critical, as a material may be scientifically elegant yet clinically impractical if it requires complex preparation, invasive delivery, unstable storage, or poor compatibility with existing clinical procedures. Therefore, digestive disease biomaterials should be designed in accordance with actual clinical workflows. Oral formulations may be more suitable for chronic maintenance therapy and repeated administration (Mikhail and Bekaii-Saab, 2015), whereas rectal formulations may be appropriate for distal colitis and localized mucosal inflammation (Xiao et al., 2016). Endoscopic spraying or injection can be used for visible and localized lesions. Perioperative application may support anastomotic protection. Implantable or adhesive systems may be developed for fistula or ulcer repair, and image-guided local therapy may be considered for gastrointestinal tumors (Quan et al., 2025). This workflow-centered approach is essential for translational gastrointestinal bioengineering because it links material design directly to practical biomedical application. It also ensures that material innovation is evaluated not only by laboratory performance, but also by feasibility, usability, reproducibility, procedural compatibility, and clinical relevance.

### 7.3 Safety, degradation, and microbiome compatibility

The gastrointestinal tract is highly sensitive to material safety. Degradation products may affect epithelial cells, immune cells, microbiota, and systemic metabolism (Byrne et al., 2021). Long-term or repeated exposure is especially relevant for chronic diseases such as IBD. Materials should therefore be evaluated not only for cytotoxicity but also for mucosal irritation, immune activation, microbial imbalance, systemic absorption, and effects on epithelial renewal.

For microbiome-modulating materials, safety assessment must include ecological consequences. A material that suppresses inflammation but destabilizes microbial diversity may create long-term risk. Conversely, microbiome-supportive biomaterials may offer broader benefits if their effects are predictable and controllable.

### 7.4 Validation of AI-guided biomaterial intervention

AI models used to guide biomaterial therapy must be validated across populations, institutions, disease subtypes, and data modalities. Bias is a major concern. Endoscopic images, microbiome datasets, and clinical records may differ by geography, diet, equipment, treatment access, and disease phenotype. A model trained in one population may not generalize to another (Ray, 2025).

For this reason, the integration of AI and biomaterials should be evidence-driven. Models should provide interpretable outputs, uncertainty estimates, and clinically meaningful decision thresholds. Biomaterial trials should prospectively test whether data-guided patient selection improves outcomes compared with conventional selection.

## 8 Future directions

Future research in gastrointestinal microenvironment reprogramming may focus on several complementary directions that extend beyond current material design and digital monitoring approaches.

First, materials will become more disease-state responsive. Instead of releasing drugs at a fixed rate, future systems may respond to combinations of pH, enzymes, ROS, cytokines, microbial metabolites, and mechanical cues.

Second, digital medicine may move from passive monitoring toward therapeutic guidance. AI models should not only detect lesions, but also help prioritize which local biological process, such as inflammation, barrier disruption, dysbiosis, fibrosis, or oxidative stress, should be targeted.

Third, endoscopy may potentially become a therapeutic bioengineering platform. In addition to visualization and biopsy, it may enable the localized delivery of hydrogels, nanoparticles, tissue adhesives, regenerative factors, or microbiome-modulating systems directly to diseased sites.

Fourth, digital twins may help simulate intervention strategies before treatment. A patient-specific model could compare systemic biologics, local biomaterial therapy, microbiome modulation, or combination strategies.

Fifth, regulatory science will become increasingly important. Combination products involving materials, drugs, biologics, devices, software, and data platforms will require new validation pathways. Translational success will depend on safety, manufacturability, reproducibility, clinical usability, and health-system integration.

## 9 Discussion

Digestive disease care is entering a period in which microenvironmental biology, biomaterials engineering, artificial intelligence, and digital medicine are beginning to converge. However, convergence should not mean simply placing these technologies side by side. The central value of the proposed framework is that it links patient-specific microenvironmental interpretation with local therapeutic execution. This connection distinguishes gastrointestinal microenvironment reprogramming from isolated advances in AI-based diagnosis or biomaterial drug delivery.

This Perspective proposes “gastrointestinal microenvironment reprogramming” as a conceptual framework for future research. Compared with conventional drug delivery, this framework emphasizes dynamic tissue context, disease-state specificity, and local biological regulation (Zhang et al., 2025). Compared with conventional digital health, it links data interpretation to physical therapeutic execution. In this model, intelligent biomaterials serve as active interfaces between biological tissues and digital decision systems.

Across inflammatory bowel disease, mucosal injury, colorectal cancer-associated inflammation, and postoperative gastrointestinal healing, the common principle is to match the dominant pathological microenvironment with an appropriate local intervention strategy. This disease-state-based logic may be more flexible than applying a single material platform to an entire diagnostic category.

Nevertheless, major barriers remain. Many biomaterial platforms are still evaluated in simplified preclinical systems. Many AI models remain disconnected from therapeutic decision-making. Digital twins for gastrointestinal disease are still largely conceptual and require rigorous validation (Sel et al., 2025). Regulatory pathways for combined material-digital interventions are complex. Most importantly, clinical adoption will require evidence that these systems improve meaningful outcomes rather than merely adding technological complexity.

The next stage of research should therefore prioritize clinically grounded questions. Which gastrointestinal microenvironmental features are most actionable? Which digital biomarkers reliably identify these features? Which material functions are most likely to change disease trajectory? Which delivery routes are practical for repeated or localized use? Which endpoints best reflect successful microenvironmental reprogramming? Answering these questions will require collaboration among gastroenterologists, biomaterials scientists, bioengineers, computational scientists, microbiome researchers, endoscopists, surgeons, and regulatory experts.

In conclusion, this Perspective proposes gastrointestinal microenvironment reprogramming as a conceptual framework for precision intervention in digestive diseases. By linking digital interpretation of patient-specific disease states with local therapeutic execution by intelligent biomaterials, this framework may help shift digestive disease care toward more proactive, adaptive, and microenvironment-oriented management.

## Data Availability

The original contributions presented in the study are included in the article/supplementary material, further inquiries can be directed to the corresponding author.
